# Supplementary material for: BUB-1 targets PP2A:B56 to regulate chromosome congression during meiosis I in C. elegans oocytes
Source: eLife. 2020 Dec 23;9:e65307. doi: 10.7554/eLife.65307 (PMC7787666; doi:10.7554/eLife.65307)
Supplement: Supplementary file 1. — Created with Clustal Omega version 2.1. [file elife-65307-supp1.docx]

|  | **PPTR-1** | **B56β1** | **B56β2** | **B56ε2** | **B56ε3** | **B56ε1** | **B56α2** | **B56α1** | **PPTR-2** | **B56δ1** | **B56δ2** | **B56δ3** | **B56γ4** | **B56γ5** | **B56γ1** | **B56γ2** | **B56γ3** |
| --- | --- | --- | --- | --- | --- | --- | --- | --- | --- | --- | --- | --- | --- | --- | --- | --- | --- |
| **PPTR-1** |  | 60.12 | 60.08 | 66.67 | 71.87 | 66.59 | 68.30 | 63.35 | 52.26 | 54.81 | 56.10 | 60.17 | 60.42 | 57.75 | 64.48 | 63.27 | 59.27 |
| **B56β1** | 60.12 |  | 97.56 | 72.17 | 73.66 | 71.83 | 71.79 | 69.77 | 56.36 | 62.75 | 63.85 | 67.13 | 65.28 | 64.47 | 66.21 | 67.13 | 64.44 |
| **B56β2** | 60.08 | 97.56 |  | 71.86 | 73.66 | 71.52 | 71.79 | 69.28 | 56.59 | 62.53 | 63.62 | 67.13 | 64.78 | 63.97 | 65.68 | 66.59 | 63.94 |
| **B56ε2** | 66.67 | 72.17 | 71.86 |  | 99.74 | 99.78 | 78.21 | 77.49 | 59.87 | 66.16 | 67.60 | 70.49 | 67.62 | 67.81 | 69.14 | 69.88 | 68.75 |
| **B56ε3** | 71.87 | 73.66 | 73.66 | 99.74 |  |  | 79.80 | 79.80 | 63.94 | 71.36 | 71.36 | 71.36 | 73.70 | 72.38 | 72.82 | 73.70 | 72.38 |
| **B56ε1** | 66.59 | 71.83 | 71.52 | 99.78 |  |  | 78.23 | 77.52 | 59.66 | 65.88 | 67.28 | 70.12 | 67.10 | 67.27 | 68.58 | 69.30 | 68.19 |
| **B56α2** | 68.30 | 71.79 | 71.79 | 78.21 | 79.80 | 78.23 |  | 99.07 | 60.84 | 67.92 | 69.14 | 69.14 | 71.53 | 69.56 | 70.74 | 71.78 | 69.79 |
| **B56α1** | 63.35 | 69.77 | 69.28 | 77.49 | 79.80 | 77.52 | 99.07 |  | 56.49 | 63.35 | 64.97 | 68.00 | 66.74 | 66.37 | 68.35 | 69.30 | 67.49 |
| **PPTR-2** | 52.26 | 56.36 | 56.59 | 59.87 | 63.94 | 59.66 | 60.84 | 56.49 |  | 62.31 | 61.76 | 66.19 | 63.55 | 60.66 | 68.97 | 66.25 | 62.74 |
| **B56δ1** | 54.81 | 62.75 | 62.53 | 66.16 | 71.36 | 65.88 | 67.92 | 63.35 | 62.31 |  | 99.65 | 98.19 | 79.07 | 75.68 | 81.29 | 80.00 | 76.72 |
| **B56δ2** | 56.10 | 63.85 | 63.62 | 67.60 | 71.36 | 67.28 | 69.14 | 64.97 | 61.76 | 99.65 |  | 98.39 | 79.72 | 77.37 | 82.46 | 81.05 | 77.63 |
| **B56δ3** | 60.17 | 67.13 | 67.13 | 70.49 | 71.36 | 70.12 | 69.14 | 68.00 | 66.19 | 98.19 | 98.39 |  | 84.40 | 81.72 | 87.62 | 85.71 | 81.72 |
| **B56γ4** | 60.42 | 65.28 | 64.78 | 67.62 | 73.70 | 67.10 | 71.53 | 66.74 | 63.55 | 79.07 | 79.72 | 84.40 |  | 94.57 | 93.00 | 93.81 | 93.61 |
| **B56γ5** | 57.75 | 64.47 | 63.97 | 67.81 | 72.38 | 67.27 | 69.56 | 66.37 | 60.66 | 75.68 | 77.37 | 81.72 | 94.57 |  | 92.87 | 94.64 | 95.23 |
| **B56γ1** | 64.48 | 66.21 | 65.68 | 69.14 | 72.82 | 68.58 | 70.74 | 68.35 | 68.97 | 81.29 | 82.46 | 87.62 | 93.00 | 92.87 |  | 99.77 | 98.44 |
| **B56γ2** | 63.27 | 67.13 | 66.59 | 69.88 | 73.70 | 69.30 | 71.78 | 69.30 | 66.25 | 80.00 | 81.05 | 85.71 | 93.81 | 94.64 | 99.77 |  | 99.79 |
| **B56γ3** | 59.27 | 64.44 | 63.94 | 68.75 | 72.38 | 68.19 | 69.79 | 67.49 | 62.74 | 76.72 | 77.63 | 81.72 | 93.61 | 95.23 | 98.44 | 99.79 |  |

**Supplementary Table 1.** Percent Identity (%) Matrix of the full length sequence alignment of mammalian B56 isoforms and *C.elegans* orthologues PPTR-1 and PPTR-2 .Created with Clustal Omega version 2.1.
