# Supplementary material for: BUB-1 targets PP2A:B56 to regulate chromosome congression during meiosis I in C. elegans oocytes
Source: eLife. 2020 Dec 23;9:e65307. doi: 10.7554/eLife.65307 (PMC7787666; doi:10.7554/eLife.65307)
Supplement: Supplementary file 3. [file elife-65307-supp3.docx]

| Markers / *mutants* | Alelle | Primers (5'-3') | primer name | Restriction enzyme |
| --- | --- | --- | --- | --- |
| *bub-1(L282A;V285A);*  *bub-1(L282A;V285A);(K718R;D847N)* | *bub-1(syb1936[bub-1((L282A;V285A)])I;*  *bub-1(syb1936,syb2383[bub-1 (L282A;V285A,K718R;D847N)]I* | ATGGAATATCGATTGAGGAGTTTC | fgp_153 | *NsiI* |
|  |  | CAATTTCGGGTTGTACTTCAGA | fgp_154 |  |
| *bub-1(S283A)* | *bub-1(syb2396[bub-1((S283A)])I* | ATGGAATATCGATTGAGGAGTTTC | fgp_153 | *HhaI* |
|  |  | CAATTTCGGGTTGTACTTCAGA | fgp_154 |  |
| *bub-1(K718R;D847N)* | *bub-1(syb1746[bub-1((K718R;D847N)])I* | GATGTGTCTTGTGCGTCCTC | fgp_125 | *BmrI* |
|  |  | CCAGTCCGGAAGTGAATCA | fgp_126 |  |
| *pptr-2Δ* | *pptr-2(ok1467)V* | ATCCTTGCTAAGCACAGTTGAAGT | fgp_137 |  |
|  |  | CGTGAACGTGACTTTCTGAAGA | fgp_138 |  |
| *san-1Δ* | *san-1(ok1580) I* | GAAACTGCACGCTTAAAGCTTG | fgp_139 |  |
|  |  | CTTTCCACGTTTCCCGTATC | fgp_140 |  |
|  |  | GGGTGATTCGGCAGAAGA | fgp_141 |  |
| PPTR-1::GFP | *pptr-1(lt89[pptr-1::gfp])V* | CCTCGGAGAGTACACAAGACC | fgp_158 |  |
|  |  | TGGAGTTGTCCCAATTCTTGTT | fgp_2 |  |
|  |  | AGCAGCAGAAGACGAGAGAGT | fgp_159 |  |
| PPTR-2::GFP | *pptr-2(lt91[pptr-2::gfp])V* | ACGCCCTCAAGATGTTCAT | fgp_160 |  |
|  |  | TGGAGTTGTCCCAATTCTTGTT | fgp_2 |  |
|  |  | ACTGGGTAAGGAAGTCGAATCA | fgp_161 |  |
| AID::GFP::GSP-2 | *gsp-2(syb545[AID::gfp::gsp-2])III* | TCTAATTGGGTTGTTTGAGCG | fgp_53 |  |
|  |  | TGGAGTTGTCCCAATTCTTGTT | fgp_2 |  |
|  |  | TTTTCCCTGGTTTGGATCC | fgp_54 |  |

**Supplementary Table 3.** List of primers used for genotyping.
